# CD8^+^ T cells Are Preferentially Activated during Primary Low Dose *Leishmania major* Infection but Are Completely Dispensable during Secondary Anti-*Leishmania* Immunity

**DOI:** 10.1371/journal.pntd.0003300

**Published:** 2014-11-20

**Authors:** Ifeoma B. Okwor, Ping Jia, Zhirong Mou, Chukwunonso Onyilagha, Jude E. Uzonna

**Affiliations:** 1 Department of Medical Microbiology, University of Manitoba, Winnipeg, Manitoba, Canada; 2 Department of Immunology, University of Manitoba, Winnipeg, Manitoba, Canada; Philadelphia Research and Education Foundation, United States of America

## Abstract

We previously showed that CD8^+^ T cells are required for optimal primary immunity to low dose *Leishmania major* infection. However, it is not known whether immunity induced by low dose infection is durable and whether CD8^+^ T cells contribute to secondary immunity following recovery from low dose infection. Here, we compared primary and secondary immunity to low and high dose *L. major* infections and assessed the influence of infectious dose on the quality and magnitude of secondary anti-*Leishmania* immunity. In addition, we investigated the contribution of CD8^+^ T cells in secondary anti-*Leishmania* immunity following recovery from low and high dose infections. We found that the early immune response to low and high dose infections were strikingly different: while low dose infection preferentially induced proliferation and effector cytokine production by CD8^+^ T cells, high dose infection predominantly induced proliferation and cytokine production by CD4^+^ T cells. This differential activation of CD4^+^ and CD8^+^ T cells by high and low dose infections respectively, was imprinted during *in vitro* and *in vivo* recall responses in healed mice. Both low and high dose-infected mice displayed strong infection-induced immunity and were protected against secondary *L. major* challenge. While depletion of CD4^+^ cells in mice that healed low and high dose infections abolished resistance to secondary challenge, depletion of CD8^+^ cells had no effect. Collectively, our results show that although CD8^+^ T cells are preferentially activated and may contribute to optimal primary anti-*Leishmania* immunity following low dose infection, they are completely dispensable during secondary immunity.

## Author Summary

It is known that CD8^+^ T cells are important for primary immunity to low dose *L. major* infection, but whether low dose-induced immunity is long lasting and whether CD8^+^ T cells are also important for memory immune response to low dose *L. major* is unknown. We studied whether infectious dose affects primary anti-*Leishmania* immunity and the contribution of CD8^+^ T cells in immunity following recovery from low and high dose infections. We found that low and high dose infections preferentially induced proliferation and cytokine production by CD8^+^ and CD4^+^ T cells, respectively, during early and late stages of infections. Also, both low and high dose-infected mice were solidly protected against secondary *L. major* challenge. Depletion of CD4^+^ cells in mice that healed low and high dose infections abolished resistance to secondary challenge, but depletion of CD8^+^ cells had no effect. Together, our results show that although CD8^+^ T cells are selectively activated and contribute to optimal primary immunity after low dose infection, they are not required for secondary immunity. This research further enhances our understanding of the immunobiology of cutaneous leishmaniasis.

## Introduction

The spectrum of disease collectively known as leishmaniasis continues to be a major threat to global health in many regions of the world. According to the World Health Organization (WHO) estimate, about 15–20 million people are afflicted with the disease and close to 2 million new cases occur annually [1]. Despite intensive research, there is currently no effective licensed vaccine for prevention of human leishmaniasis. This is in part related to lack of proper understanding of the immunobiology of the disease, particularly the factors that regulate the induction, maintenance and loss of protective immunity. Because *Leishmania* are obligate intracellular parasites, a strong T cell-mediated immunity is critical for effective control of the infection. Indeed, T cell deficient mice are highly susceptible to *Leishmania* infection, and adoptive transfer of T cells restores resistance in these mice [2]. Although it is widely believed that CD4^+^ T cells are the key lymphocyte subset that regulates anti-*Leishmania* immunity, studies utilizing low dose infections show that CD8^+^ T cells are also important for optimal primary immunity [3], [4]. Thus, while CD8 deficient mice are still resistant to high dose *L. major* infection, low dose infection of these mice results in uncontrolled parasite proliferation and impaired IFN-γ response [3]. However, no study has addressed the impact of parasite dose on the magnitude of initial T cell (both CD4^+^ and CD8^+^) expansion, and whether this affects the development of secondary anti-*Leishmania* immunity. In addition, the contribution of CD8^+^ T cells to low dose infection-induced resistance is not known.

In this present study, we compared the primary and secondary immune responses to low and high dose *L. major* infections in mice. In particular, we investigated whether parasite dose affects the quality (subset) and magnitude of the initial (primary) T cell expansion and the impact of this on infection-induced (secondary) anti-*Leishmania* immunity. In addition, we also assessed the role of CD8^+^ T cells in secondary immunity following primary low dose *L. major* infection. We show that the early immune response to primary low dose *L. major* infection is dominated by CD8^+^ T cells while high dose infection preferentially induced proliferation and IFN-γ production by CD4^+^ T cells. Interestingly, mice that healed their primary low and high dose infections displayed comparable delayed-type hypersensitivity (DTH) response and rapid parasite control following secondary *L. major* challenge. Depletion of CD4^+^ cells in mice that healed their low and high dose infections led to impaired parasite control following secondary challenge. In contrast, depletion of CD8^+^ cells had no effect on control of secondary *L. major* challenge.

## Materials and Methods

### Ethics Statement

All experiments in this study were reviewed and approved by University of Manitoba Animal Care and Use Committee. Protocol #: 12-072. The University of Manitoba Animal Care and Use Committee adhere to the guidelines and standards stipulated by the Canadian Council for Animal Care.

### Mice

Six to eight weeks old female C57BL/6 mice were purchased either from Charles River Laboratory, St. Constante, Quebec or from the University of Manitoba Central Animal Care Services (CACS) breeding facility. Six to eight weeks old female C57BL/6 (Thy1.1) mice were purchased from The Jackson Laboratories (Bar Harbor, ME). All mice were maintained in specific-pathogen free environment at the CACS facility.

### Parasites

*Leishmania major* parasites (MHOM/IL/80/Friedlin) were grown in Graces' Insect medium (Invitrogen, Life Technologies, Burlington, Ontario, Canada) supplemented with 20% heat inactivated fetal bovine serum (FBS), 2 mM L glutamine, 100 U/ml penicillin, 100 µg/ml streptomycin, 25 mM HEPES (complete parasite medium). All media additives were purchased from Invitrogen. Seven day stationary phase promastigotes were used for all infection.

### Primary Infection, Secondary Challenge, and Measurement of DTH Response

Groups of C57BL/6 mice (4–6 mice per group) were infected with 1×10^3^ (low lose) or 2×10^6^ (high dose) parasites suspended in 50 µl (footpad infection) of sterile PBS. For secondary challenge, infected mice were challenged with 5×10^6^ parasites in the contralateral footpads between 12–16 weeks following primary infection when lesions were fully resolved. Lesion development and delayed type hypersensitivity response (DTH) following primary and secondary challenge infections, respectively, were determined by measuring the thickness of infected footpads with Vernier caliper (Fisher Scientific, Ottawa, ON Canada).

### Determination of Parasite Burden

At different times after primary infection or 3 weeks after secondary challenge, mice were sacrificed and parasite burden in the footpads was determined by limiting dilution as previously described [5]. Briefly, the footpads were collected and homogenized in 2 ml complete parasite medium using 15 ml tissue grinders (VWR, Edmonton, AB, Canada). The suspension was then plated in 96-well plates in triplicates at 10-fold serial dilution, incubated for 7 days at 27°C and assessed for parasite growth under a microscope.

### Adoptive Transfer and *In Vivo* Recall Response

Draining lymph-node (dLN) and spleen cells from healed (>12 weeks post-infection) donor (Thy1.2) mice infected with low or high dose *Leishmania major* were labeled with CFSE dye as described previously [6] and transferred into naïve Thy1.1 recipient mice by intravenous injection (30 million cells per recipient mouse). Twenty-four hours after adoptive transfer, recipient mice were infected with 5 million *L. major*. After 7 days, mice were sacrificed and dLN cells were assessed for proliferation and cytokine (IFN-γ and TNF) secretion directly *ex vivo* by flow cytometry following 5 hr. *in vitro* stimulation with phorbol myristic acetate (PMA; 50 ng/ml), ionomycin (500 ng/ml), and brefeldin A (BFA, 10 µg/ml).

### Bone Marrow–Derived Dendritic Cells (BMDC), *In Vitro* Cultures and Co-culture Experiments

BMDCs were generated from naïve mice and infected with *Leishmania major* promastigotes as described previously [7]. At sacrifice, the draining lymph nodes (dLNs) were harvested and made into single cell suspensions. In some experiments, the dLN cells were labeled with CFSE dye and cultured in 96-well round bottom plates (2×10^5^/well in 200 µl) in the presence of soluble *Leishmania* antigen (SLA, 50 µg/ml) as described previously [6]. In some experiments, the cells were co-cultured with infected BMDCs for 4 days (at BMDC:lymph node cell ratio of 1∶100). At the end of the culture period, the cells were stimulated with PMA (50 ng/ml), ionomycin (500 ng/ml) and BFA, (10 µg/ml) for 4 hours and routinely stained for CD4, CD3, CD8, IFN-γ and TNF and analyzed by flow cytometry.

### Depletion of T Cells

CD4^+^ and CD8^+^ T cells were depleted in healed mice 24 hr. before *L. major* challenge by injection of 500 µg of anti-CD4 (GK1.5) and anti-CD8 (TIB 210) monoclonal antibody intraperitoneally. Previous studies from the lab show that this dose of antibody leads to complete depletion (>98%) of CD4^+^ and CD8^+^ positive cells, respectively, for up to 7 days.

### *Ex vivo* Staining of Dendritic Cells

High and low dose *L. major* infected mice were sacrificed at days 7 and 14 weeks post-infection and the draining lymph nodes collected, minced into small pieces and digested with 2 ml RPMI-1640 medium containing 2% FBS, 1 mg/ml collagenase Type 1 A (Sigma, Oakville ON, Canada) and 100 µg/ml DNase I (Roche, Mississauga ON, Canada) by incubating for 25 minutes at 37°C. The tissues were further disrupted with a tissue grinder and the suspension was filtered through a 70 µm cell strainer (Roche) to remove tissue debris, and washed with 10 ml complete medium by centrifuging at 1200 rpm for 5 minutes. The cell pellets were resuspended in 2 ml complete medium, counted, stained directly *ex vivo* with different flourochrome-conjugated mAbs against CD11c, CD40, CD86, CD103, CD8α and MHC II and analyzed by flow cytometry.

### *Ex vivo* Assessment of Memory T Cell Subsets

High and low dose *L. major* infected mice were sacrificed at 14 weeks post-infection and single cell suspensions of the draining lymph nodes and spleens were made. The cell were counted and adjusted to 5×10^6^/ml and 100 µl aliquots were stained directly *ex vivo* with different flourochrome-conjugated mAbs against CD3, CD4, CD8, CD44 and CD62L and analyzed by flow cytometry.

### Statistical Analysis

Student T test was used to compare mean and standard error of mean (SEM) between two groups. In some experiments, nonparametric one-way analysis of variance (ANOVA) was used to compare mean and standard deviation (SD) of more than two groups. Tukeys test was used where there was significant difference in ANOVA. Differences were considered significant when p<0.05.

## Results

### Kinetics of Lesion Development and Cell Recruitment Following High and Low Dose *L. major* Infection

**Figure 1:**
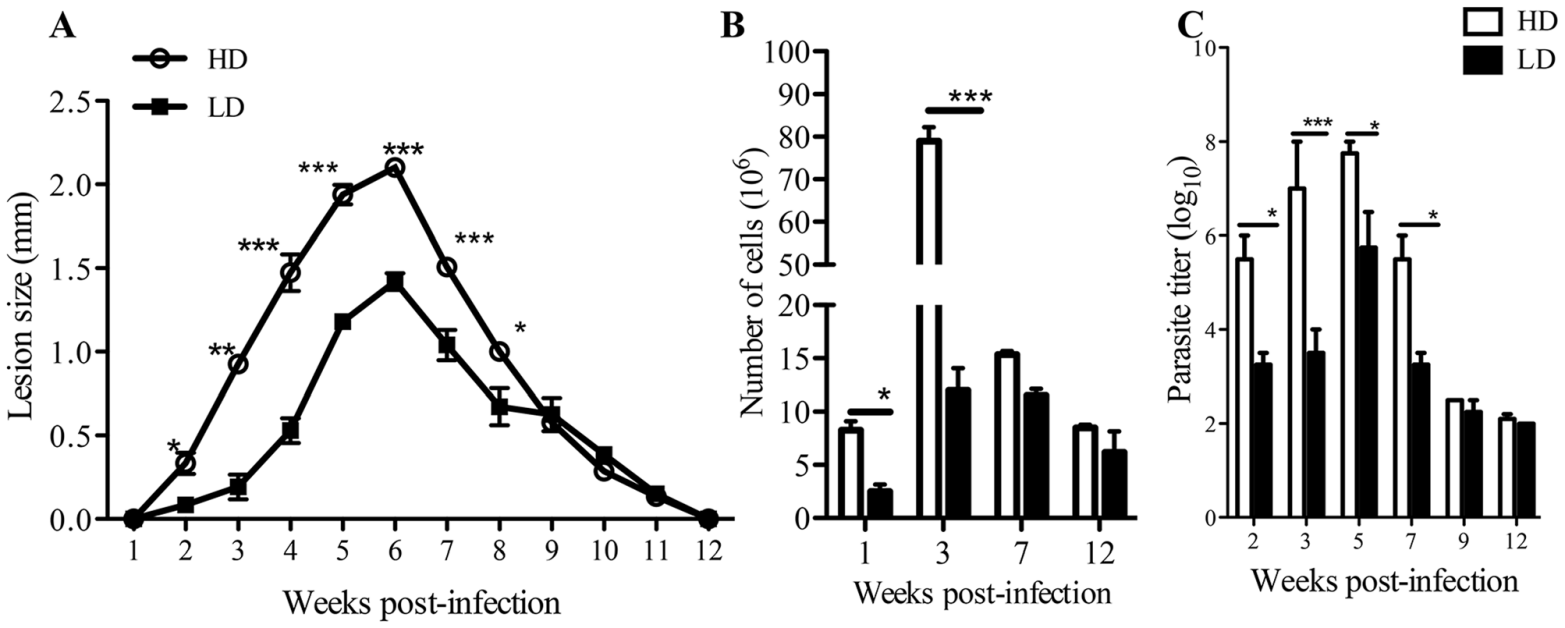
Kinetics of lesion development, cell recruitment in the draining lymph nodes and parasite burden following high and low dose *L. major* infections. C57BL/6 mice were infected with low (1×10^3^) or high (2×10^6^) dose *L. major* promastigotes in their right hind footpad and lesion size was measured weekly with Vernier callipers (A). At indicated times, some mice were sacrificed and the number of cells in the draining lymph nodes (dLNs) was determined by cell counting (B). Parasite burden in the infected footpads was determined by limiting dilution assay (C). Results presented are representative of 3 independent experiments (n = 4 mice/group) with similar results. *, p<0.05; **, p<0.01; ***, p<0.001.

We infected mice with low or high dose *L. major* and at indicated times sacrificed them to determine parasite burden and number of cells in the draining lymph nodes (dLNs). The pattern of lesion development in the footpads was similar in both low and high dose-infected mice although the overall swelling sizes were significantly (p<0.05–0.001) higher in mice infected with high dose parasites from 2–7 weeks post-infection (Fig. 1A). In addition, high dose-infected mice had significantly (p<0.05–0.001) more cells in their dLNs at 2 and 3 weeks post-infection than those infected with low dose parasites (Fig. 1B). Beyond 3 weeks, there was no significant difference in the number of cells in the dLNs, despite continued difference in lesion size that lasted up to 8 weeks post-infection (Fig. 1A). Parasite burden in the lesions of high dose-infected mice was also significantly (p<0.05–0.001) higher at 2, 3, 5 and 7 weeks post-infection than in low dose-infected mice but this difference was absent by 9 weeks post-infection (Fig. 1C). These results show that although the size of cutaneous lesion, inflammation and cell recruitment into the dLN are different following high and low dose *L. major* infections, the pattern and time to lesion resolution and parasite clearance are comparable.

### Differential Expansion and Induction of T Cell Subsets after Low and High Dose *L. major* Infection

**Figure 2:**
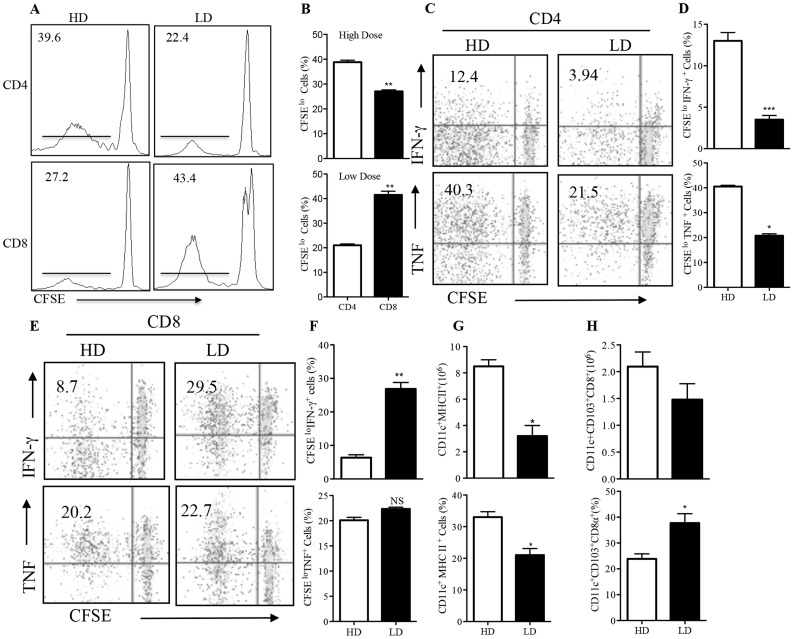
High and low dose infections preferentially expand CD4^+^ and CD8^+^ T cells, respectively. C57BL/6 mice were infected with low (1×10^3^) or high (2×10^6^) dose *L. major* promastigotes in their right hind footpad, sacrificed after 7 days and the draining lymph-node cells were labelled with CFSE dye and co-cultured with *L. major*-infected bone marrow-derived dendritic cells (BMDC) at a DC: lymph node cell ratio of 1∶100. After 4 days, the percentages of proliferating (CFSE^lo^) CD4^+^ and CD8^+^ T cells (A and B) were assessed by flow cytometry. (A) is a representative histogram plots of individual animals while B represents the mean +/− SE of proliferating cells of all the animals (6 mice) within the group. Proliferating (CFSE^lo^) IFN-γ (upper panels) and TNF (lower panels) -producing CD4^+^ (C and D) and CD8^+^ (E and F) T cells were also determined by flow cytometry. C and E are representative dot plots of individual animals within the group while D and F represent the mean +/− SE of proliferating cytokine-producing cells of all the animals (6 mice) within the group. The lymph nodes draining the infected feet were collected and digested with collagenase and the cells were then stained with different fluorochrome-conjugated antibodies against different DC subsets and the absolute numbers (upper panel) and percentage (lower panel) of CD11c^+^MHCII^+^ (G) and CD11c^+^CD103^+^CD8α^+^ (H) dendritic cells were determined by flow cytometry. Live cells were first gated on CD11c^+^ cells and further analyzed for MHC class II, CD103 and CD8α expression. Results are representative of 2 independent experiments (n = 6 mice/group) with similar results. *, p<0.05; **, p<0.01; ***, p<0.001.

CD8^+^ T cells play important role in optimal immunity to primary low dose *L. major* infection but are dispensable for immunity high dose infections [3]. To determine the influence of parasite dose on the early expansion and activation of different T cell subsets following *L. major* infection, we co-cultured CFSE-labeled dLN cells from mice infected with low and high dose *L. major* (one week post-infection) with *L. major*-infected BMDCs and assessed T cell proliferation and cytokine (IFN-γ and TNF) production by flow cytometry. There was a striking difference in proliferation of CD4^+^ and CD8^+^ T cells from dLNs of low and high dose-infected mice with high dose infection inducing significantly (p<0.05–0.01) more CD4^+^ T cell proliferation compared to low dose infections (Fig. 2A and B). Conversely, low dose infection induced significantly (p<0.05–0.01) more CD8^+^ T cell proliferation than high dose infection (Fig. 2A and B). Consistent with this, the percentage (Fig. 2C and D) and mean fluorescence intensity (MFI, Fig. S1A and S1B) of IFN-γ and TNF-producing CD4^+^ T cells in dLNs of high dose-infected mice were higher than those of low dose-infected mice. In contrast, the percentage and MFI of IFN-γ and TNF-producing CD8^+^ T cells were significantly (p<0.01) higher in low dose-infected mice (Fig. 2E and F, Fig. S1C and S1D). Consistent with the higher CD4^+^ T cell response, the percentage and absolute numbers of MHC II^+^CD11c^+^ (dendritic) cells in dLNs of high dose-infected mice were significantly (p<0.05) higher than those of low dose-infected mice (Fig. 2G). Interestingly, the percentage of CD11c^+^CD103^+^CD8α^+^ dendritic cells was higher in the dLNs of low dose than in high dose-infected mice (Fig. 2H). However, there was no significant difference in the expression of key costimulatory molecules (including CD86 and CD40) on dendritic cells from high and low dose-infected mice (Fig. S2A and S2B).

### The Differential Expansion of T Cells during Early Low and High Dose Infection Is Sustained throughout Infection

**Figure 3:**
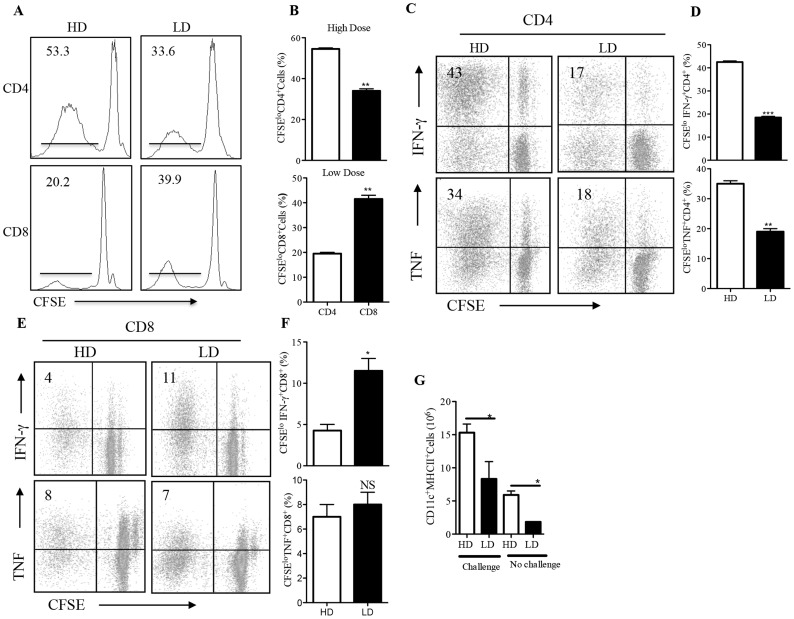
The differential expansion of CD4^+^ and CD8^+^ T cells by high and low dose infections, respectively is sustained during recall response. C57BL/6 mice infected with low dose (1×10^3^) and high dose (2×10^6^) *L. major* and allowed to completely resolve (heal) their lesion (>12 wks.). Healed mice were sacrificed and dLN cells were labelled with CFSE dye and co-cultured with *L. major*-infected BMDCs at a DC:dLN cell ratio of 1∶100. After 4 days, the percentages of proliferating (CFSE^lo^) CD4^+^ and CD8^+^ T cells were determined by flow cytometry (A and B). The percentages of IFN-γ (upper panel) and TNF (lower panel) -producing CD4^+^ (C and D) and CD8^+^ (E and F)) T cells within the proliferating (CFSE^lo^) population were also assessed. Shown are the representative histogram (A) and dot (C and E) plots of individual animals within the group and bar graphs (B, D and F) showing the means +/− SE of all the animals (3–4 mice) within the group. The absolute numbers of CD11c^+^MHCII^+^ dendritic cells in the draining lymph nodes of high and low dose infected mice were also determined by flow cytometry following Collagenase/Dispase digestion (G). Results presented are representative of 3 independent experiments (n = 3–4 mice/group) with similar results. *, p<0.05; **, p<0.01; ***, p<0.001.

Next, we wished to determine whether the early differential expansion of CD4^+^ and CD8^+^ T cells subsets by high and low dose *L. major* infection, respectively, is transient and related to differences in parasite burden at the infection site (see Fig. 1C). Therefore, we assessed T cell responses (proliferation and cytokine production) after 12 weeks when lesion is fully resolved and parasite burden in the footpads and dLNs of high and low dose-infected mice are comparable (see Fig. 1C). Flow cytometric analyses show that akin to observations during early infection, the percentage of proliferating CD4^+^ T cells was significantly (p<0.01) higher in high dose-infected mice compared to the low dose infected group (Fig. 3A and B). In contrast, there were significantly (p<0.01) more proliferating CD8^+^ T cells in the low dose-infected mice compared to the high dose-infected mice (Fig. 3A and B). Furthermore, the percentage of proliferating and IFN-γ and TNF-secreting CD4^+^ T cells in mice infected with high dose parasites were significantly (p<0.05) higher than those from low dose-infected mice (Fig. 3C and D). In contrast, mice that healed their low dose infection had significantly (p<0.05) higher percentage of proliferating and cytokine- (IFN-γ and TNF) secreting CD8^+^ T cells than those that healed high dose infection (Fig. 3E and F). In addition, and consistent with observations during early infection, the absolute numbers of dendritic cells (CD11c^+^) expressing MHC II were significantly (p<0.05) higher in healed high dose-infected mice compared to those from healed low dose-infected mice (Fig. 3G). Collectively, these results suggest that an early antigen encounter creates an imprint in T cell subset responses that is maintained throughout the course of *L. major* infection.

### Differential Expansion of T Cell Subsets in Low and High Dose Infected Mice Occurs *In Vivo*

**Figure 4:**
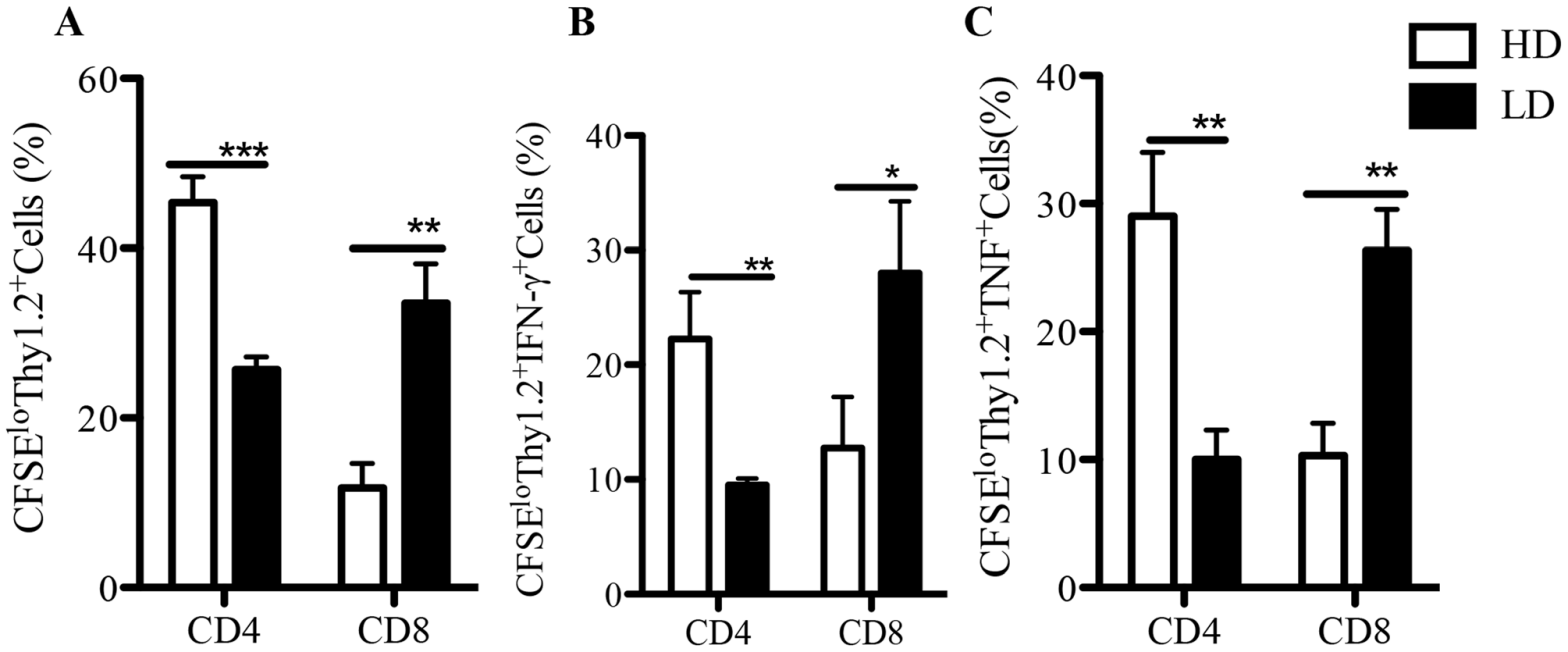
Differential expansion of CD4^+^ and CD8^+^ T cells following high and low dose infections with *L. major* occur *in vivo*. Thy1.2 C57BL/6 mice that healed their primary low dose (1×10^3^) or high dose (2×10^6^) *L. major* infections were sacrificed and their spleen cells were labeled with CFSE dye and adoptively transferred (3×10^7^) to Thy1.1 congenic recipient mice. After 24 hr, the recipient mice were challenged with 5×10^6^ parasites, sacrificed after 1 wk and the percentages of proliferating donor (CFSE^lo^) Thy1.2^+^ (donor) cells (A) were determined by flow cytometry. In addition, the percentages of proliferating (CFSE^lo^) IFN-γ (B) and TNF (C) -producing donor (Thy1.2^+^) cells were also determined by flow cytometry. Results presented are representative of 2 independent experiments (n = 3–4 mice/group) with similar results. *, p<0.05; **, p<0.01; ***, p<0.001.

To confirm that the preferential activation of CD8^+^ and CD4^+^ T cell responses by low and high dose infections, respectively, is physiologic and occurs *in vivo*, we adoptively transferred CFSE-labeled CD3^+^ cells isolated from Thy1.2 mice that healed either low or high dose *L. major* infection into naïve Thy1.1 recipient mice. Recipient mice were challenged 24 hr. later with *L. major*, sacrificed after one week and donor (Thy1.2^+^) cells from the dLNs were assessed directly *ex vivo* for proliferation and cytokine (IFN-γ and TNF) production by flow cytometry. As shown in Fig. 4, donor cells from low dose-infected mice contain significantly (p<0.05) higher percentage of proliferating (CFSE^lo^) CD8^+^ cells whereas those from high dose-infected mice had significantly (p<0.05) higher percentage of proliferating CD4^+^ cells (Fig. 4A). Similar to the *in vitro* findings, the percentage of proliferating IFN-γ and TNF-producing CD8^+^ T cells were significantly (p<0.05) higher in cells from low dose-infected donor mice compared to those from high dose-infected donor mice (Fig. 4B and C). In contrast, donor cells from high dose-infected mice had significantly (p<0.05) more proliferating and cytokine-producing CD4^+^ cells than those from low dose-infected mice (Fig. 4B and C). Collectively, these results show that high and low dose *L. major* infections-induced differential T cell responses occur *in vivo* and create an imprint that is maintained over time during the course of infection.

### High and Low Dose *L. major* Infection Induce Comparable Protection against Virulent Challenge

**Figure 5:**
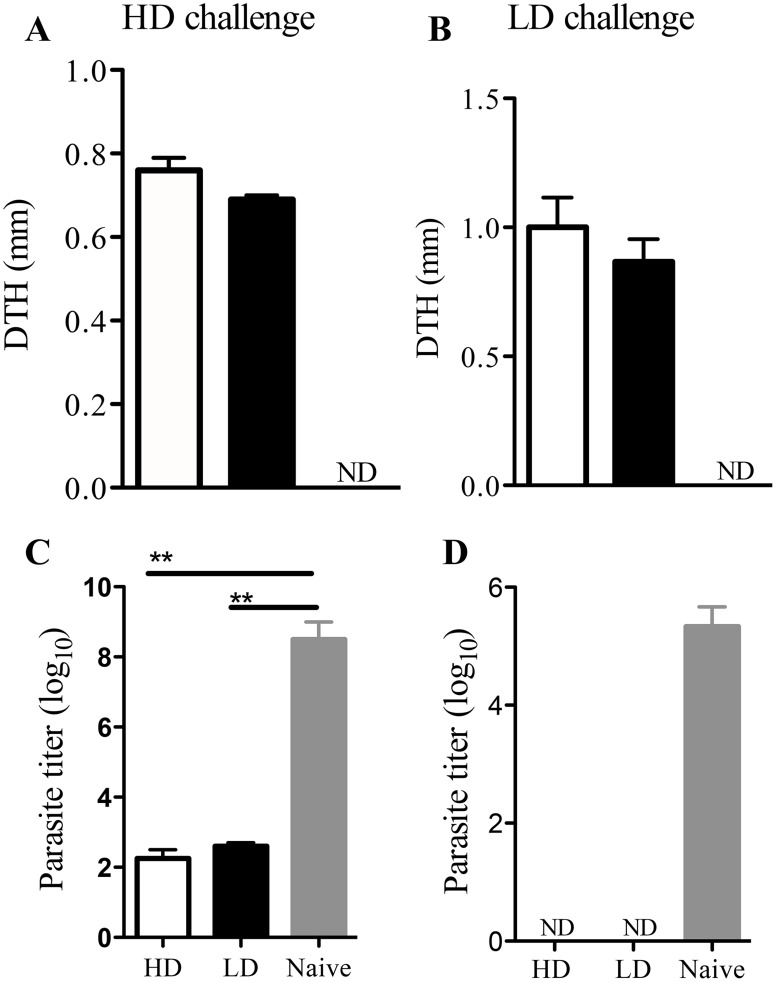
Low and high dose *L. major* infection leads to comparable protection against secondary virulent challenge. C57BL/6 mice were infected with low (1×10^3^) or high dose (2×10^6^) *L. major* and allowed to completely resolve their lesions (>12 wks.). Healed mice and some naïve age-matched controls were challenged with 5×10^6^ (high dose challenge, A and C) or 1×10^3^ (low dose challenge, B and D) *L. major* in the contra-lateral footpad and delayed-type hypersensitivity (DTH) response was determined at 72 hr post-challenge (A and B). Three weeks after challenge, mice were sacrificed and parasite burden in the challenged footpads was determined by limiting dilution assay (C and D). Results presented are representative of 3 independent experiments (n = 3–4 mice/group) with similar results. **, p<0.01. ND, not detected.

Although we found that the pattern of lesion development and parasite clearance are similar in mice infected with low and high dose *L. major*, it is possible that they induce different types or subsets of memory cells, which might affect the quality of secondary anti-*Leishmania* immunity. Indeed, previous studies show that the quality of memory T cell response is influenced in part by antigen dose [8], [9]. Interestingly, we found no significant difference in the percentages of CD44^+^CD62L^lo^ (effector memory-like, Tem) or CD44^+^CD62L^hi^ (central memory-like, Tcm) CD4^+^ and CD8^+^ T cells in the dLNs and spleens of mice that healed primary low and high dose *L. major* infections (Fig. S3A and S3B), suggesting that parasite dose may not influence the quality of anti-*Leishmania* memory T cell responses. In line with this, when mice that healed high or low dose primary infection were rechallenged with either high (Fig. 5A and C) or low (Fig. 5B and D) dose *L. major*, there were no significant differences in either DTH response (Fig. 5A and B) or rapid parasite control in the challenged footpads (Fig. 5C and D), suggesting that both low and high dose infections induce qualitatively comparable infection-induced resistance in healed mice.

### CD8+ T Cells Are Dispensable for Protection against Secondary Leishmania Major Infection

**Figure 6:**
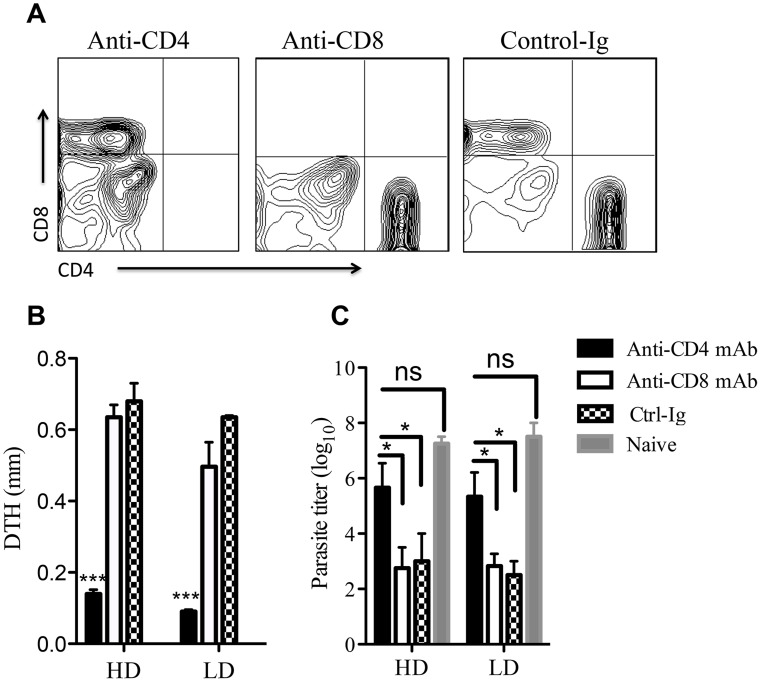
CD8^+^ T cells are dispensable for secondary anti-*Leishmania* immunity. C57BL/6 mice infected with low (1×10^3^) or high (2×10^6^) dose *L. major* were allowed to completely resolve their lesions (>12 wks.). Healed mice were treated with anti-CD4 (clone GK1.5) or anti-CD8 (clone TIB 210) mAbs to deplete CD4^+^ or CD8^+^ cells, respectively (A). Control mice received rat IgG1 (isotype control) antibody. After 24 hr., treated mice were challenged with 5×10^6^ *L. major* and DTH response was measured at 72 hr. post-challenge (B). Three weeks after challenge, mice were sacrificed and parasite burden in the challenged footpads was determined by limiting dilution (C). Age-matched naïve mice served as controls. Results presented are representative of 2 independent experiments (n = 3–4 mice/group) with similar results. *, p<0.05; ***, p<0.001.

Although we previously reported that CD8^+^ T cells are important for optimal immunity to primary low dose *L. major* infection [3] it is not known whether they also contribute to secondary anti-*Leishmania* (infection-induced) immunity. We observed herein a preferential expansion of CD8^+^ cells during recall responses in mice that healed their low dose *L. major* infection (see Figs. 3B and 4B), suggesting that CD8^+^ T cells might also be critical for secondary anti-*Leishmania* immunity in low dose-infected mice. Therefore, we treated healed low and high dose-infected mice with anti-CD4 or anti-CD8 mAb (to deplete CD4^+^ and CD8^+^ cells, respectively, Fig. 6A) and challenged them after 24 hr. with either high or low dose *L. major*. Surprisingly, depletion of CD8^+^ cells in both high and low dose-infected mice did not affect DTH response (Fig. 6B) and rapid parasite control (Fig. 6C) following high dose challenge. In contrast, CD4^+^ T cell depletion in both low and high dose-infected mice resulted in significant (p<0.05) impairment in DTH (Fig. 6B) and rapid parasite control (Fig. 6C). Similar results were also obtained following low dose *L. major* challenge of low or high dose healed mice following CD4^+^ or CD8^+^ T cell depletion (Fig. S4A and S4B). Collectively, these results indicate that although low dose *L. major* infection preferentially expands CD8^+^ T cells, CD4^+^ T cells are the major players that mediate secondary anti-*Leishmania* immunity in mice. They further show that although CD8^+^ T cells are important for optimal immunity to primary low dose infection [3], they are completely dispensable during a secondary challenge.

## Discussion

Clinical observations and experimental studies suggest that the development of effective cell-mediated immunity is essential for protection against leishmaniasis. However, the lack of a universally approved and effective vaccine against human leishmaniasis suggests that we still do not completely understand the factors that regulate the development of cell-mediated immunity against the disease. Although CD4^+^ T cells are critical for protective immunity in cutaneous leishmaniasis [10], CD8^+^ T cells have also been shown to be essential in certain situations, particularly in low dose infections [3], [11]. During low dose *L. major* infection, CD8^+^ T cells were shown to contribute to lesion resolution and parasite control by producing IFN-γ that augment optimal CD4^+^ Th1 response [3], [11]. However, whether CD8^+^ T cells also contribute to secondary anti-*Leishmania* immunity following resolution of primary infection is unclear. In addition, no study has investigated the impact of low parasite dose on secondary anti-*Leishmania* immunity. We show here that the patterns of lesion development and parasite burden were similar in both high and low dose infections, the quality of the immune response was strikingly different. Whereas high dose infection induced strong CD4^+^ T cell proliferation and Th1 cytokine response, low dose infection predominantly activates CD8^+^ T cells. Using adoptive transfer of T cells from healed mice into naïve congenic recipients, we also demonstrated that this differential activation of CD4^+^ and CD8^+^ T cells by high and low dose infections, respectively, is observed *in vivo* following *L. major* challenge. Interestingly, while depletion of CD4^+^ T cells in mice that healed both high and low dose infections led to loss of immunity following secondary *L. major* challenge, depletion of CD8^+^ T cells had no effect. Taken together, the results presented here show that although low dose *L. major* infection preferentially activates CD8^+^ T cells that contribute to optimal primary immunity, they are completely dispensable for resolution of secondary *L. major* challenge.

Previous studies showed that CD8^+^ T cells produce large amounts of IFN-γ following *L. major* infection and were critical for optimum primary immunity [3], [11]. In addition, earlier studies suggest that CD8^+^ T cells are activated following secondary *L. major* challenge, suggesting that they contribute to secondary anti-*Leishmania* immunity [12], [13]. We found that CD8^+^ cells are completely dispensable for protective secondary anti-*Leishmania* immunity. Healed mice (following primary low dose or high dose infections) depleted of CD8^+^ T cells before virulent low or high dose rechallenge were as resistant as those treated with control-Ig. This is an agreement with our previous observation which showed that CD8 deficient mice that healed their primary high dose infection are resistant to virulent *L. major* challenge [3]. However, whether CD8^+^ T cells are critical for secondary immunity following high or low dose challenge in mice that were exposed to primary low dose infection has never been reported. In this current study, we addressed this question by depleting CD8^+^ T cells in mice that healed their primary low dose infection before being challenged with either low or high dose virulent parasites. We show that regardless of the dose at both primary and secondary challenges, CD8^+^ T cells are completely dispensable during secondary anti-*Leishmania* immunity (Fig. 6C and D). In contrast, depletion of CD4^+^ T cells led to complete loss of infection-induced immunity. Collectively, these observations suggest that the role of CD8^+^ T cells may be limited to helping for optimal activation of CD4^+^ Th1 cells during primary infection. Once effective CD4^+^ Th1 response is induced, CD8^+^ T cells are no longer relevant, thus making them dispensable during a secondary response. We believe that differences in animal models, parasite strain and experimental design could account for the discrepancy between our findings and the studies that found a role for CD8^+^ T cells in secondary immunity. For example, in those studies, splenocytes were first depleted of CD4^+^ cells and then cultured *in vitro* for extended period of time before assessing for IFN-γ production by CD8^+^ T cells [12], [13]. Such *in vitro* culture conditions could potentially influence the magnitude of CD8^+^ T cell responses that otherwise would not be seen in short-term and/or bulk whole cell cultures as performed in our study.

Why would high and low dose infections differentially activate CD4^+^ and CD8^+^ T cells, respectively? It is conceivable that this may be related in part to differences in activation threshold for CD4^+^ and CD8^+^ T cells. It has been shown that naïve CD4^+^ T cells require at least 6 hours of contact with APCs presenting their cognate peptides in order to acquire optimum signals leading to activation, proliferation and cytokine production [14]. In contrast, naïve CD8^+^ T cells require less than 2 hours of antigenic stimulation to acquire enough signals required for their activation, proliferation and cytokine release, suggesting that the requirements for activating CD8^+^ T cells are less stringent [15]. Hence, low dose infection provides lower antigen availability that favors activation of CD8^+^ T cells. In contrast, high infectious dose provides high antigen load that could overcome the need for longer contact thus favoring expansion of CD4^+^ T cells. In addition, high dose infection was associated with strong upregulation of MHC class II molecules on DCs (Fig. 2G), which would potentially favor activation CD4^+^ T cells. Further more, we found that the percentage of CD103^+^CD8α^+^ dendritic cells in the draining lymph nodes of low dose-infected mice were significantly higher than those in high dose-infected mice. Given that CD103^+^CD8α^+^ dendritic cells cross present exogenous antigens to CD8^+^ T cells leading to their activation and effector cytokine response [16], [17], it is conceivable that the higher of induction of this subset of dendritic cells contributes to preferential activation of CD8^+^ T cells following low dose infection. Although differences in co-stimulatory molecules expression has been associated with differences in activation of CD4^+^ and CD8^+^ T cells [18], it is unlikely that these contributed to differential induction of CD4^+^ and CD8^+^ T cells in our model system. In line with this, we did not observe any difference in expression of CD40 and CD86 molecules on DCs from the dLNs following low and high dose infections, suggesting that the effect of antigen dose is mostly restricted to TCR-peptide interaction and not on co-stimulation.

Comparing the lesion outcome and parasite burden in mice infected with high and low dose *L. major* infection, we found that although high dose infection was associated with significantly higher lesion size and parasite burden early during infection, disease resolution (healing) occurred almost at the same time and this was associated with comparable level of protection following virulent challenge. This finding, which is in agreement with our previous report [3] has important implications in vaccine development as well as vaccination strategies against cutaneous leishmaniasis. Following recovery from natural *L. major* infection (which is usually self-resolving), a long-term (sometimes lifelong) immunity develops to reinfection. This observation is the basis for leishmanization, which is the deliberate inoculation of lesion-derived virulent parasites into hidden parts of the body in order to prevent a more serious visible cutaneous disease. Leishmanization is the oldest and only effective preventive practice against human cutaneous leishmaniasis [19]. The usual practice in leishmanization is to employ a relatively high dose inoculum because it is believed that such high dose is able to induce strong inflammatory and immune responses necessary for protection against subsequent reinfections. As a result, some leishmanized individuals develop large (sometimes non-healing) lesions that require medical treatment. In some cases, exacerbated chronic skin disease and/or immunosuppression have been reported [20]. Due to the effectiveness of leishmanization, recent efforts have focused on ways to make the practice safer, including suggestions to include killed parasites in the inoculum [21] or to use genetically engineered attenuated parasites [22]. Whether high doses of parasites during leishmanization (as is currently practiced) is required for protection is unclear. We show here that despite inducing comparatively lesser inflammatory responses (smaller lesion sizes), low dose infection induced secondary immunity and protection following virulent *L. major* challenge comparable to high dose infection. This observation indicates that vaccination with large dose of live virulent parasite is not necessary to achieve protection. They further suggest that leishmanization with low dose inoculum could be a viable alternative practice as it would lead to smaller lesion at the inoculation site that is less prone to ulceration and secondary bacteria infection. In line with this, it has been shown that low dose infection of the highly susceptible BALB/c mice leads to resistance and protection against virulent *L. major* challenge [23]. Collectively, our study shows that parasite dose critically influence the magnitude of expansion of CD4^+^ and CD8^+^ T cells following *L. major* infection. While low dose infection preferentially activates CD8^+^ T cells, high dose infection leads to preferential activation of CD4^+^ T cells. Surprisingly, despite the strong activation of CD8^+^ T cells and their importance in primary immunity following low dose infection, secondary immunity in mice that healed their low dose *L. major* infection was completely dependent on CD4^+^ (and not CD8^+^) T cells.

## Supporting Information

Figure S1 **Mean fluorescence intensity of IFN-γ and TNF-producing CD4^+^ and CD8^+^ T cells after following low and high dose** ***L. major*** **infection.** C57BL/6 mice were infected with low dose (1×10^3^) or high (2×10^6^) dose *L. major* parasites in their right hind footpad. Seven days after infection, mice were sacrificed and the draining lymph-node cells were labeled with CFSE dye and co-cultured for 4 days with *L. major*-infected bone marrow-derived dendritic cells (BMDC) at a DC:lymph node cell ratio of 1∶100. The cells were then routinely stained for surface molecules (CD3, CD4 and CD8) and intracellular cytokine (IFN-γ and TNF) expression and analyzed by flow cytometry. Shown are bar graphs representing the Mean Fluorescence Intensity (MFI) of proliferating (CFSE^lo^) and IFN-γ (A and C) and TNF (B and D) producing CD4^+^ cells (A and B) CD8^+^ (C and D) cells. Results are representative of 2 independent experiments (n = 6 mice/group) with similar results. *, p<0.05; **, p<0.01; ***, p<0.001.
(TIF)

Figure S2 **Comparable expression of costimulatory molecules on lymph node dendritic cells following primary low and high dose** ***L. major*** **infection.** Naïve C57BL/6 mice were infected with low (LD, 1×10^3^) or high (HD, 2×10^6^) dose *L. major* parasites and after 7 days sacrificed and the lymph nodes draining the infected feet were collected and digested with collagenase. The cells were then stained with fluorochrome-conjugated antibodies and the expression of CD86 and CD40 by CD11c^+^ cells was analyzed by flow cytometry. Shown are the absolute numbers of CD11c^+^CD86^+^ (A) and CD11c^+^CD40^+^ (B) dendritic cells in the lymph nodes of LD and HD infected mice.
(TIF)

Figure S3 **Low and high dose** ***L. major*** **infection induces comparable memory-like T cell subsets.** C57BL/6 mice were infected with low dose (10^3^) or high dose (2×10^6^) *L. major* and allowed to completely resolve their lesions (>12 wks.). Healed mice were sacrificed and the percentage of central memory-like (CD44^hi^CD62L^hi^) and effector memory-like (CD44^hi^CD62L^lo^) cells within CD4^+^ (upper panels) and CD8^+^ (bottom panels) T cell populations in the draining lymph nodes (A) and spleens (B) were determined by flow cytometry. Results presented are representative of 2 independent experiments (n = 3–4 mice/group) with similar results.
(TIF)

Figure S4 **CD8^+^ T cells are dispensable for secondary anti-** ***Leishmania*** **immunity following low dose challenge.** C57BL/6 mice infected with low (1×10^3^) or high (2×10^6^) dose *L. major* were allowed to completely resolve their lesions (>12 wks.). Healed mice were treated with anti-CD8 (clone TIB 210) mAbs to deplete CD8^+^ cells. Control mice received rat IgG1 (isotype control) antibody. After 24 hr., treated mice were challenged with 1×10^3^ *L. major* and DTH response was measured at 72 hr. post-challenge (A). Three weeks after challenge, mice were sacrificed and parasite burden in the challenged footpads was determined by limiting dilution (B). Age-matched naïve mice served as controls. Results presented are representative of 2 independent experiments (n = 3–4 mice/group) with similar results. ND, not detected.
(TIF)
