# Supplementary figures and images for: CD8+ T cells Are Preferentially Activated during Primary Low Dose Leishmania major Infection but Are Completely Dispensable during Secondary Anti-Leishmania Immunity
Source: PLoS Negl Trop Dis. 2014 Nov 20;8(11):e3300. doi: 10.1371/journal.pntd.0003300 (PMC4238992; doi:10.1371/journal.pntd.0003300)

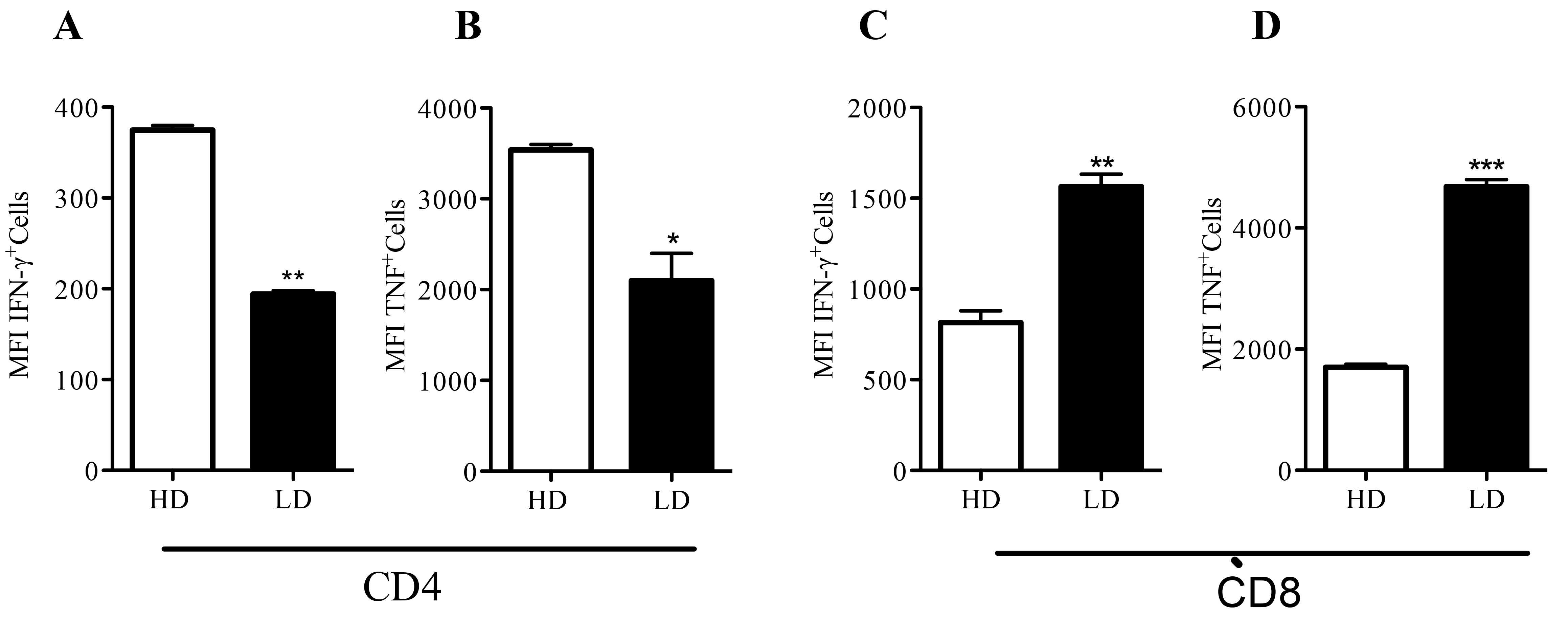

Supplement: Figure S1 — Mean fluorescence intensity of IFN-γ and TNF-producing CD4+ and CD8+ T cells after following low and high dose L. major infection. C57BL/6 mice were infected with low dose (1×10^3^) or high (2×10^6^) dose L. major parasites in their right hind footpad. Seven days after infection, mice were sacrificed and the draining lymph-node cells were labeled with CFSE dye and co-cultured for 4 days with L. major-infected bone marrow-derived dendritic cells (BMDC) at a DC:lymph node cell ratio of 1∶100. The cells were then routinely stained for surface molecules (CD3, CD4 and CD8) and intracellular cytokine (IFN-γ and TNF) expression and analyzed by flow cytometry. Shown are bar graphs representing the Mean Fluorescence Intensity (MFI) of proliferating (CFSElo) and IFN-γ (A and C) and TNF (B and D) producing CD4+ cells (A and B) CD8+ (C and D) cells. Results are representative of 2 independent experiments (n = 6 mice/group) with similar results. *, p<0.05; **, p<0.01; ***, p<0.001. (TIF) [file pntd.0003300.s001.tif]

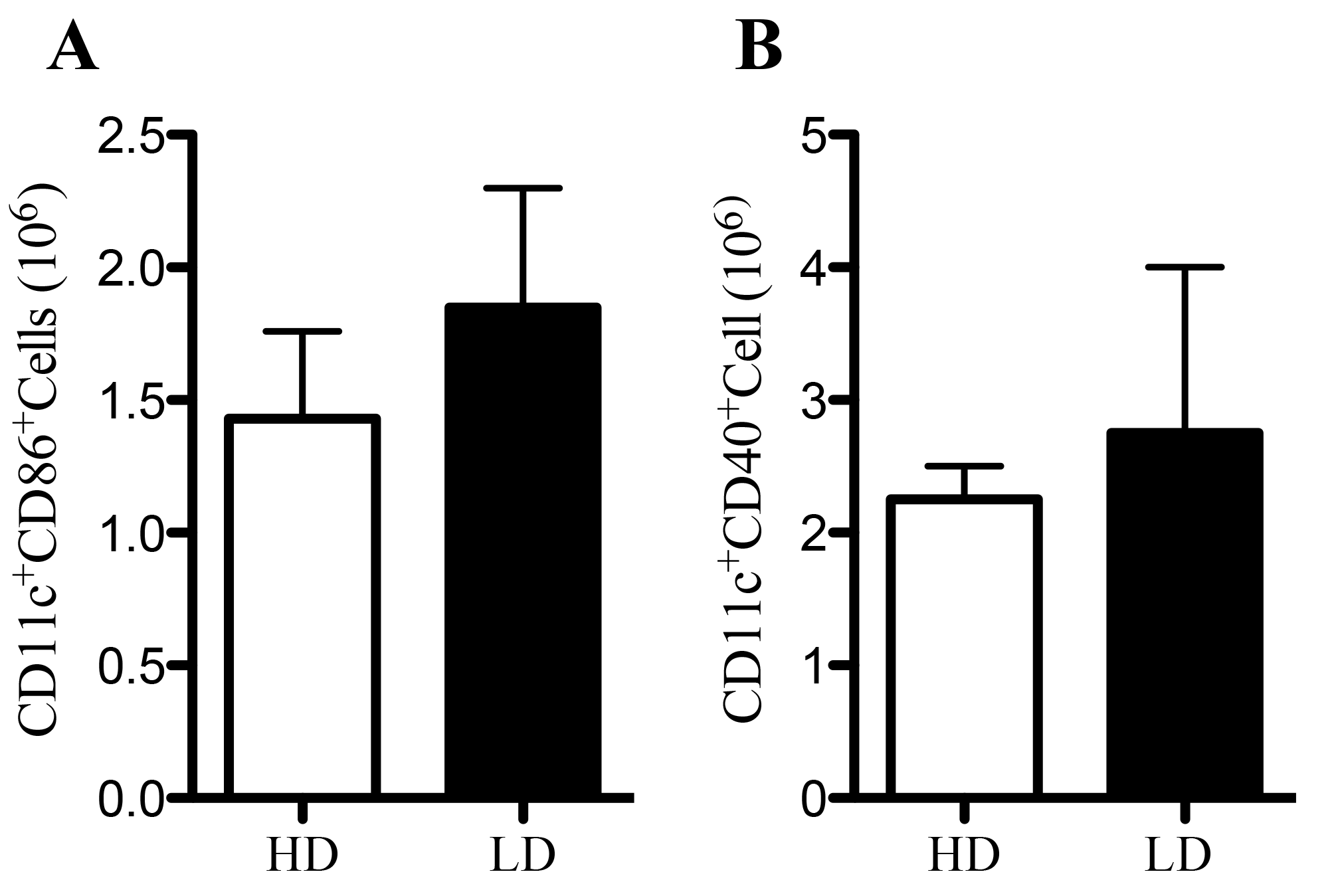

Supplement: Figure S2 — Comparable expression of costimulatory molecules on lymph node dendritic cells following primary low and high dose L. major infection. Naïve C57BL/6 mice were infected with low (LD, 1×10^3^) or high (HD, 2×10^6^) dose L. major parasites and after 7 days sacrificed and the lymph nodes draining the infected feet were collected and digested with collagenase. The cells were then stained with fluorochrome-conjugated antibodies and the expression of CD86 and CD40 by CD11c+ cells was analyzed by flow cytometry. Shown are the absolute numbers of CD11c+CD86+ (A) and CD11c+CD40+ (B) dendritic cells in the lymph nodes of LD and HD infected mice. (TIF) [file pntd.0003300.s002.tif]

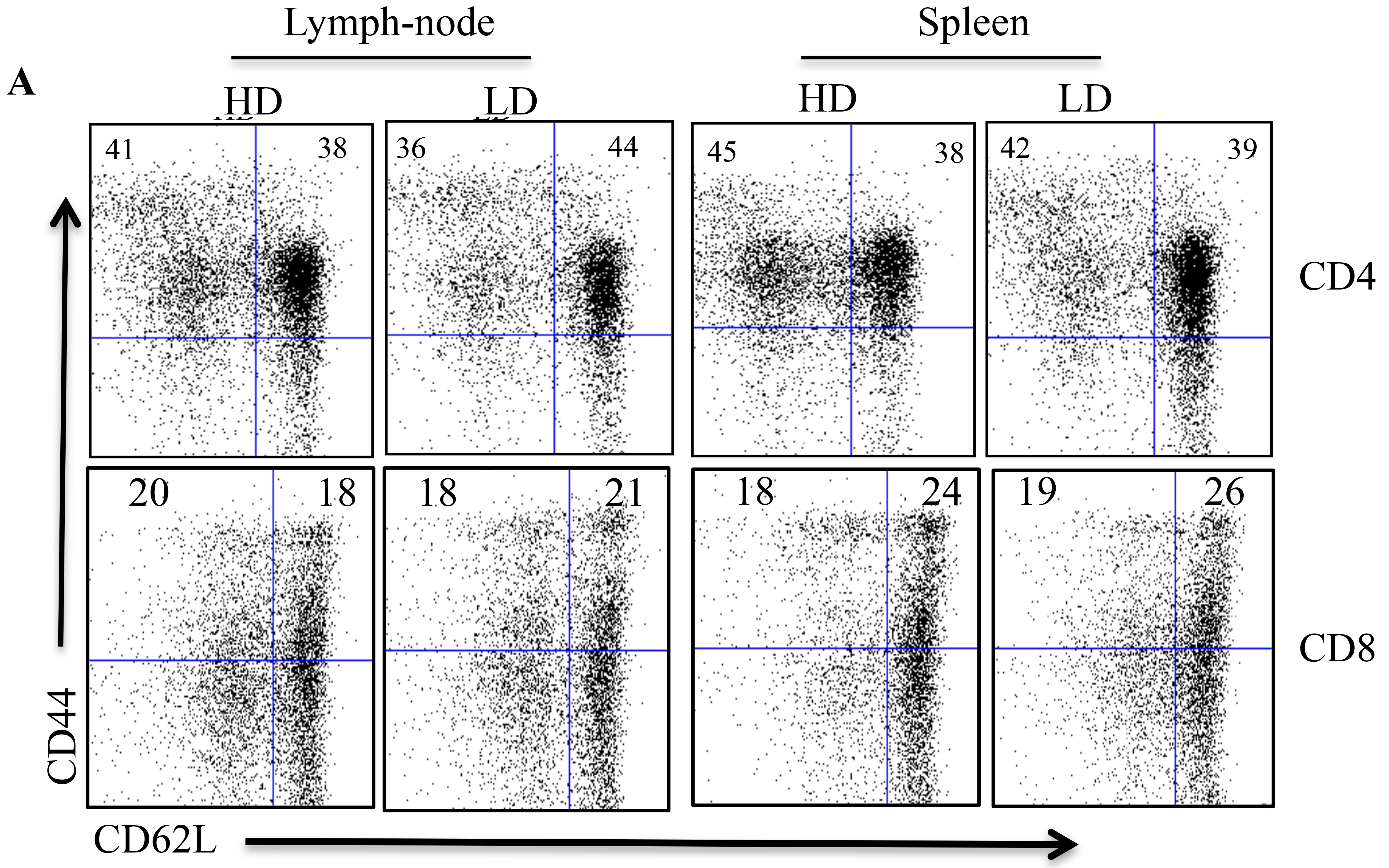

Supplement: Figure S3 — Low and high dose L. major infection induces comparable memory-like T cell subsets. C57BL/6 mice were infected with low dose (103) or high dose (2×10^6^) L. major and allowed to completely resolve their lesions (>12 wks.). Healed mice were sacrificed and the percentage of central memory-like (CD44hiCD62Lhi) and effector memory-like (CD44hiCD62Llo) cells within CD4+ (upper panels) and CD8+ (bottom panels) T cell populations in the draining lymph nodes (A) and spleens (B) were determined by flow cytometry. Results presented are representative of 2 independent experiments (n = 3–4 mice/group) with similar results. (TIF) [file pntd.0003300.s003.tif]

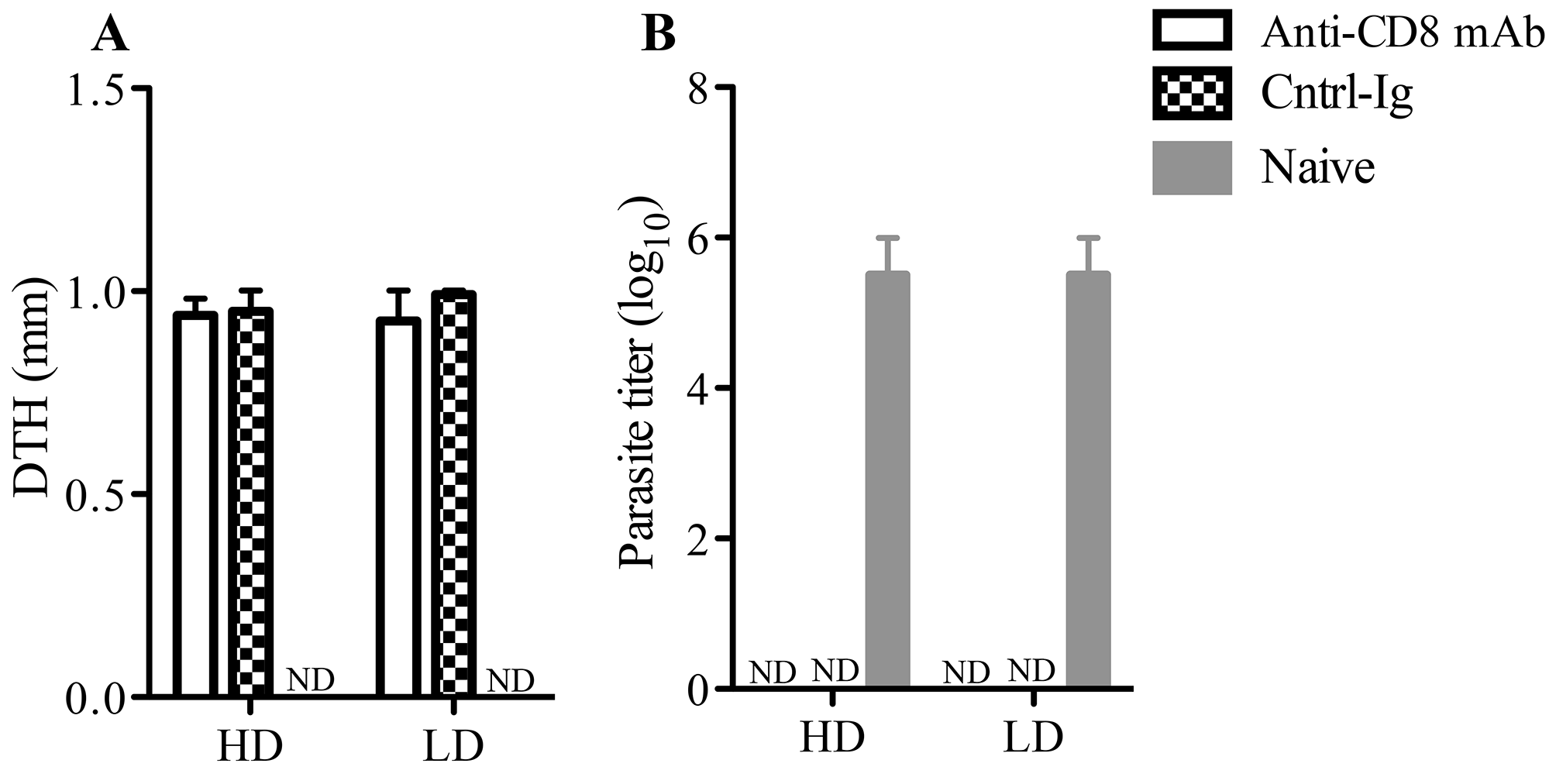

Supplement: Figure S4 — CD8+ T cells are dispensable for secondary anti- Leishmania immunity following low dose challenge. C57BL/6 mice infected with low (1×10^3^) or high (2×10^6^) dose L. major were allowed to completely resolve their lesions (>12 wks.). Healed mice were treated with anti-CD8 (clone TIB 210) mAbs to deplete CD8+ cells. Control mice received rat IgG1 (isotype control) antibody. After 24 hr., treated mice were challenged with 1×10^3^ L. major and DTH response was measured at 72 hr. post-challenge (A). Three weeks after challenge, mice were sacrificed and parasite burden in the challenged footpads was determined by limiting dilution (B). Age-matched naïve mice served as controls. Results presented are representative of 2 independent experiments (n = 3–4 mice/group) with similar results. ND, not detected. (TIF) [file pntd.0003300.s004.tif]
